# Generating Flavor Molecules Using Scientific Machine Learning

**DOI:** 10.1021/acsomega.2c07176

**Published:** 2023-03-15

**Authors:** Luana P. Queiroz, Carine M. Rebello, Erbet A. Costa, Vinícius V. Santana, Bruno C. L. Rodrigues, Alírio E. Rodrigues, Ana M. Ribeiro, Idelfonso B. R. Nogueira

**Affiliations:** † LSRE-LCM-Laboratory of Separation and Reaction Engineering-Laboratory of Catalysis and Materials, Faculty of Engineering, University of Porto, Rua Dr. Roberto Frias, 4200-465 Porto, Portugal; ‡ ALiCE-Associate Laboratory in Chemical Engineering, Faculty of Engineering, University of Porto, Rua Dr. Roberto Frias, 4200-465 Porto, Portugal; § Departamento de Engenharia Química, Escola Politécnica (Polytechnic Institute), Universidade Federal da Bahia, 40210-630 Salvador, Brazil; ∥ Chemical Engineering Department, Norwegian University of Science and Technology, Sem Sælandsvei 4, Kjemiblokk 5, 7491 Trondheim, Norway

## Abstract

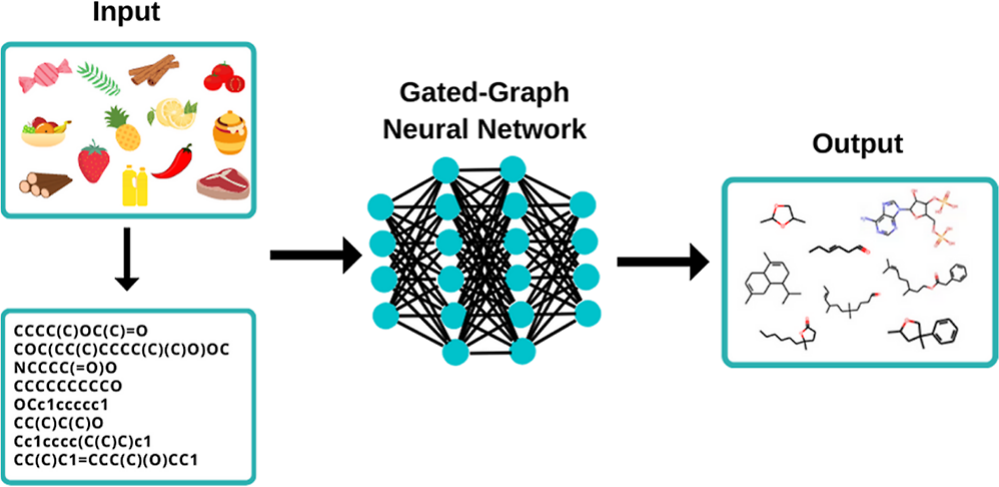

Flavor is an essential component in the development of numerous products in the market. The increasing consumption of processed and fast food and healthy packaged food has upraised the investment in new flavoring agents and consequently in molecules with flavoring properties. In this context, this work brings up a scientific machine learning (SciML) approach to address this product engineering need. SciML in computational chemistry has opened paths in the compound’s property prediction without requiring synthesis. This work proposes a novel framework of deep generative models within this context to design new flavor molecules. Through the analysis and study of the molecules obtained from the generative model training, it was possible to conclude that even though the generative model designs the molecules through random sampling of actions, it can find molecules that are already used in the food industry, not necessarily as a flavoring agent, or in other industrial sectors. Hence, this corroborates the potential of the proposed methodology for the prospecting of molecules to be applied in the flavor industry.

## 1 Introduction

The understanding and development of flavor result from two disparate but intertwined subjects, chemistry and sensory science, applied by the flavorists to develop new products.^1^ The chemical development of flavor depends on the understanding of how the chemical compounds convey flavor to the product. This is carried out by aiming to replicate their effect on the biological response. So, the underline hypothesis behind this is that there is a correlation between the chemical properties of a given compound and the provoked flavor sensation. However, the creation and replication of flavor (the engineering behind it) are complex, as it must evoke the smell and taste simultaneously, a multisensory experience.^2^ In this scenario, flavor engineering has emerged as a field of product engineering that aims to fulfill the needs of the market and consumers through the development of new flavors and flavor-based products.^3^ This is a new field that needs more profound development to supply new tools to this industry. Flavor engineering can help this sector develop new products to deal with the modern society’s healthy style while addressing several other concerns found in the industry nowadays.

Experimental studies in flavor engineering were performed by Monteiro et al. (2018).^4^ The sensory quality of flavor-based products was analyzed, alongside their psychophysical models, through chromatographic techniques. The applied methodology allowed us to evaluate dominant features of aromas and, also, a sensorial evaluation. In the same context, the work of Rodrigues et al. (2021)^3^ brings on a review of the developments in performance, classification, and the design of mixtures of fragrances and perfumes. In this review, an approach for flavor engineering is proposed, being an extension of the one for perfume engineering.

Nature has approximately 2500 flavor chemicals that can be replicated by other synthetic molecules. The recreation and analysis of these chemicals allow the discovery of synthetic flavors that are stable, cost-effectively produced, purer, and more potent. Even though the possibilities are vast, the complexity of combining the molecules that can translate the right sensation as a nerve signal is a trial-and-error process.^2^ Moreover, the flavor and flavor-based product development must consider the applicable law and regulations, the associated health issues, and the environmental damage that the synthetic chemicals process can cause.^5^ Hence, flavor development is costly and can be considerably reduced by employing new technologies. Therefore, scientific machine learning (SciML) can bring a new perspective to this process.

SciML is another emergent field that aims to adapt machine learning (ML) tools to a given application domain. It has been applied as an efficient and resource-saving method in general game playing, data mining, bioinformatics, and computational chemistry.^6^ Another important development in SciML is the implementation of this technique in mathematical physics to solve computational mechanics problems. Samaniego et al. (2020)^7^ proposed the application of SciML techniques to solve partial differential equations as an approach to solve engineering problems. The application of machine learning and computer science in chemistry has increased significantly. It is promising in designing, synthesizing, and generating molecules and materials.^8^ More specifically, a shy but increasing trend in applying ML tools can be seen in flavors.

Park et al. (2021)^9^ proposed a methodology to innovate the food industry focused on food pairing. FlavorGraph is presented as a graph embedding method to recommend food pairings based on food representations. Although it presents limitations on the food-related information available and the lack of scientific evaluation of the results obtained, the FlavorGraph presents an innovative application of deep learning in the flavor industry. Xu (2019)^10^ developed a bachelor’s thesis that combined a generative adversarial network (GAN) with a variational autoencoder (VAE) to analyze a recipe database and discover the missing ingredient of recipes. The referred work presented remarkable results in clustering recipes of the same geo-ethnic cuisine group and searching for the ingredients. Nevertheless, the presented technology cannot extract or manipulate structures since the model collapses.

Even though there are applications of SciML in the flavor engineering field, works that explore the potential of SciML in the development of new flavors and flavory molecules were not yet found. The use of SciML in this potential field can be a useful tool in the solution of the challenges already described. These tools can be used as a simple and reliable way to identify new chemical molecules that can be synthesized and considered natural. These are two specific goals that SciML can address much faster than the usual routes. It is possible to find some works in other fields that make use of SciML to prospect new elements for a given application. For example, Mercado et al. (2021)^11^ developed a platform to design molecules using deep neural network architectures, the GraphINVENT. However, in the field of flavor engineering, these tools need to be reshaped to meet this domain’s specific demands. Furthermore, new strategies need to be developed to efficiently apply these ideas in flavor engineering. For instance, the limited information regarding the flavor of chemical compounds is a challenge to consider.

The work of Zhang et al. (2021)^12^ compared numerous deep molecular generative models, including CharRNN, REINVENT, AAE, VAE, ORGAN, LatentGAN and GraphINVENT. For this study, the authors trained all the mentioned models using the GDB-13 database, a database of drug-like compounds. In terms of overall compound coverage, REINVENT was the best model, and ORGAN presented the lowest performance. The GraphINVENT method performed better than all the other deep generative models (DGMs) studied when considering the ring system and functional group coverage. This result is explained through the probabilistic sampling of actions for graph generation of this model. Meanwhile, the GAN-based models presented the worst performance in all three metrics analyzed, the ring system, functional group, and molecular coverage. This result is explained by the fact that the generator in those models is supposed to copy the true data in the adversarial training, which decreases the generalization capability. Furthermore, it is important to highlight that the alternative in the flavor field is to find new molecules by trial-and-error; therefore, developing a generative approach for this purpose is already improving the state of the art.

This work aims to develop a new standpoint in flavor engineering based on SciML. It is proposed to build a new approach to develop flavors and flavor-based products based on generative neural network models.

## 2 Methodology and Results

### 2.1 Database

The database used in the development of this work was extracted from FlavorDB’s website^13^ through a web scraper code developed for this purpose. The extracted information consisted of the PubChem ID, chemical name, flavor descriptors of the molecule, and the SMILES representation for 921 valid molecules. Moreover, a data curation step was automatically performed to ensure the quality of the database and that it only had canonical SMILES, which is described as follows.

**Figure 1:**
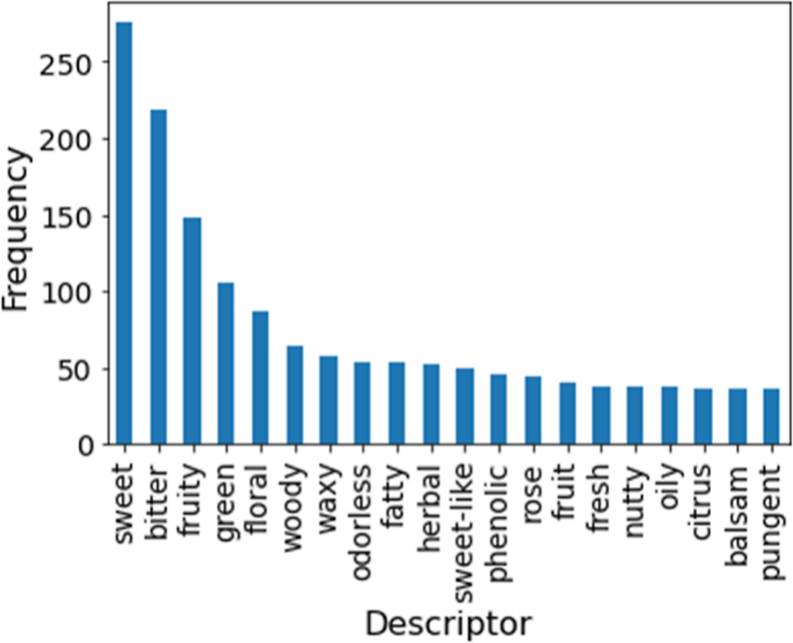
Bar plot of the 20 most common flavor’s descriptors’ frequency in the database.

An analysis of this database was performed. A total of 417 flavor descriptors were found. The five most common were sweet, bitter, fruity, green, and floral, following this order, which altogether occurred 1512 times. Figure 1 presents the database’s 20 most common descriptors’ frequencies.

**Figure 2:**
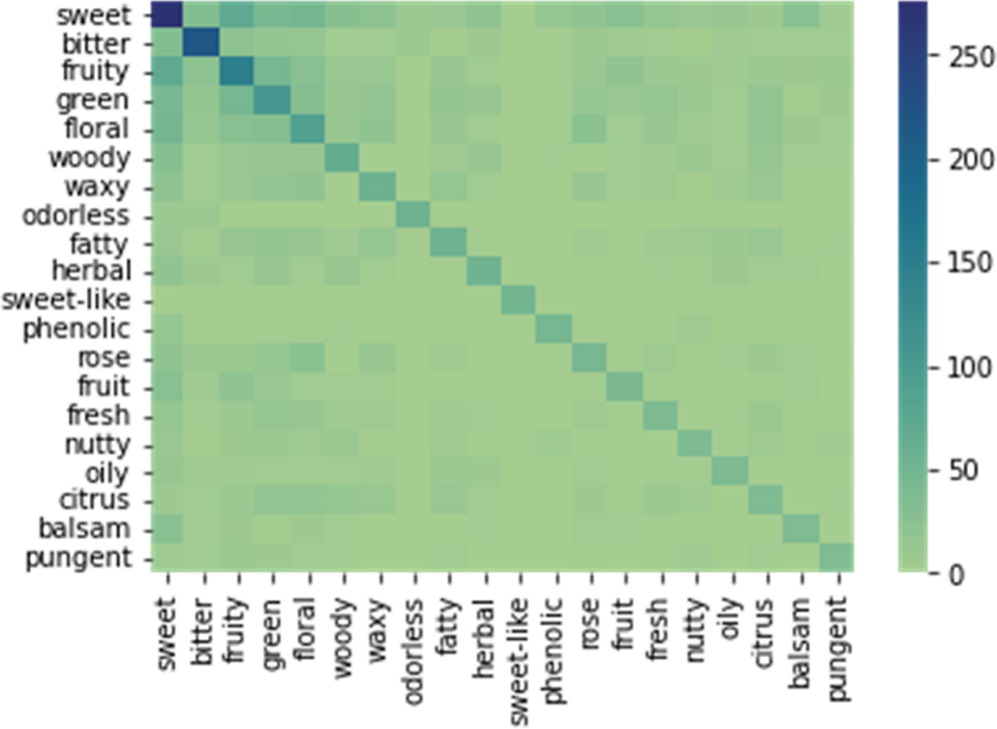
Co-occurrence heat map for the 20 most common flavor’s descriptors in the database.

A co-occurrence heat map was made, Figure 2, to understand the relation between the descriptors. This is an important tool since the same molecule can have more than one flavor descriptor associated. As shown in Figure 2, it is possible to visualize the frequency of the co-occurrence between the 20 most common descriptors. Also, it is possible to analyze how the descriptors are correlated to each other. For example, fruity co-occurs more with sweet and green. This analysis is an important tool for flavor engineering, as it provides insights into the flavor’s relationship.

With the database ready, the next step is to define the inputs of the proposed methodology. As its purpose is to create molecules, the framework input should be chemical properties, such as types of atoms, formal charge, and the maximum number of atoms present in the database. Table 1 presents the required information, which is defined following the overall chemical properties found in the database built.

**Table 1:** Chemical Property Input^a^

types of atoms	C, N, O, F, P, S, Cl, Br, I
formal charge	0
maximum number of atoms	69

a It should be pointed out that, in order to avoid the overfitting in the model, the database acquired was split into train, test, and validation sets. 60% of the database was allocated to train, following 20% for test and 20% for validation.

### 2.2 Generative Model

DGMs are a resourceful approach to identify patterns of likelihood between samples and learn a concealed or complex probability distribution from unconstrained and evenly distributed samples. The structure of the neural networks with numerous hidden layers in the DGM, if successfully trained, enables the generation of new samples with similar properties to the original ones. Originally the DGM was presented as a contestant to the traditional quantum-mechanical computation to predict properties. This deep learning technique is a cost-effective computational resource to approximate complex high-dimensional probabilities. This clears the way for new developments in cheminformatics regarding molecular science, such as the prospect of generating desired molecules.^14−16^

**Figure 3:**
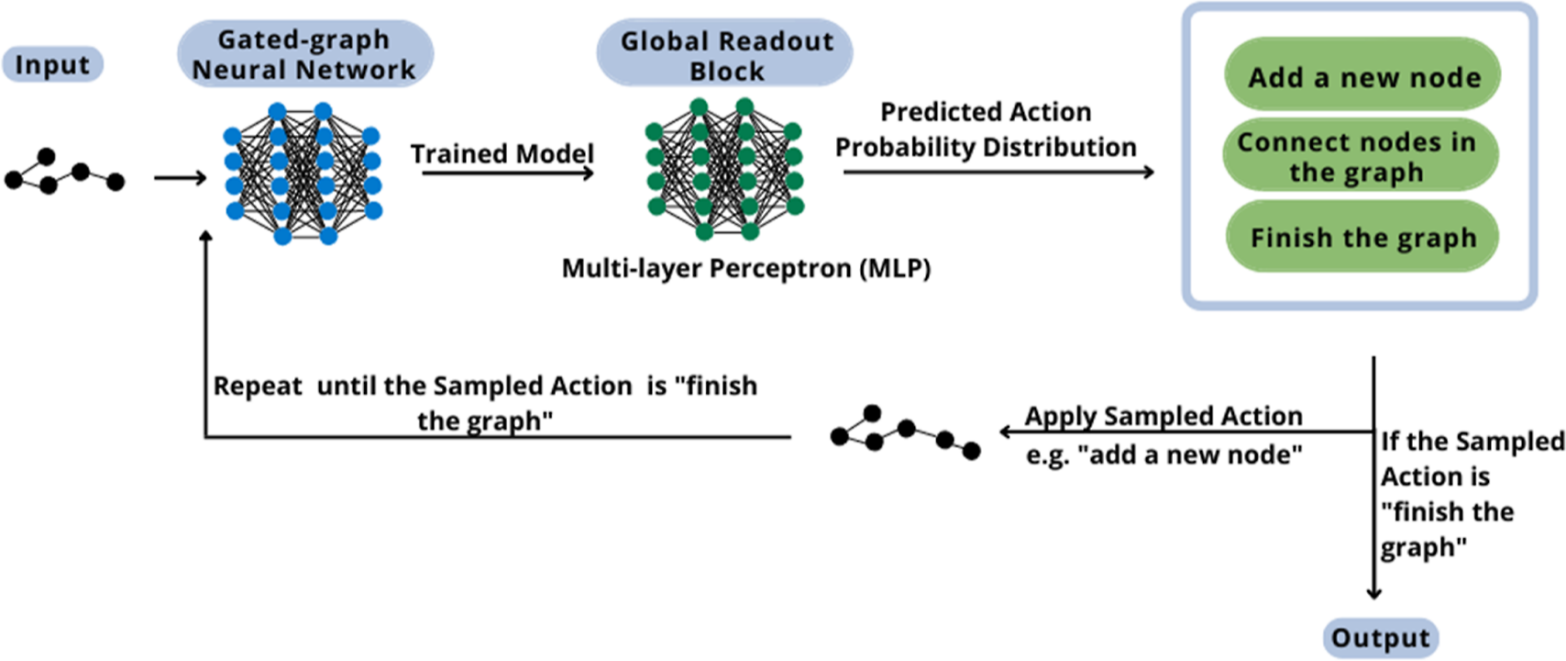
DGM methodology scheme.

The following figure, Figure 3, describes the methodology step that involves the generative model. The selection of the types of neural networks used in the methodology’s construction is based on the conclusions of the work of Mercado et al. (2021).^11^

The molecule is in the canonical SMILES form in the database, so it is necessary to convert from SMILES to molecules and from molecules to graph. In this way, the graphs can be used as the generative input. For this purpose, the RDKit functions were used, Open-Source Cheminformatics Software accessible in Python. The conversion from molecules to graphs consists of transforming atoms into nodes and bonds into edges. These nodes and edges have embeddings, in which the chemical information associated with them is stored. The embeddings make it possible to understand the relationship between the components of the graph. Hence, the generative system receives as input one molecule at a time in the form of graph. Then, the generation will proceed one bond at a time.

Additionally, the graph structure has additional features, for instance, the adjacency matrix and edge attributes. The adjacency matrix represents how the nodes are related to each other in a squared matrix with dimensions defined by the number of nodes in the graph. The edge attributes translate the distance between the edges in the graph.

The molecule in the graph form, alongside its associated features, is preprocessed so that the model can learn how to construct and deconstruct it properly. For this instance, the canonical deconstruction path is followed, similar to the one followed in the work of Mercado et al. (2021).^11^ Weininger et al. (1988)^17^ defined the canonical method that gives a unique chemical structure. In the graph form, each node (atoms) and edges (bonds) are labeled numerically according to their type. Then, the starting node is selected, and the sequential nodes’ order is defined according to the canonical labels given. The canonical deconstruction path follows the mentioned labeling and order to learn how to construct and deconstruct the molecules, aiming to learn how to generate new ones.

In the training step, the molecular graph, the adjacency tensor (*E*), and the node feature matrix (*X*) are given as the input to the gated-graph neural network (GGNN).^18,19^

The GGNN provides as the output the graph embedding (*g*) and the final transformed node feature matrix (*H*^*L*^). These outputs are the required input to the global readout block, using a multi-layer perceptron (MLP) architecture as a unique feedforward artificial neural network. The global readout block is applied to predict each graph’s action probability distribution (APD) to guide the model in the construction of the new graph.

The functioning of the data flow in the MLP is in the forward direction, from the input to the output. In this case, two hidden layers are used in the structure, and the prediction of the APD is performed by the output layer.^20,21^

The property of interest to be predicted by the MLP, the APD, consists of a vector comprising the expected probability for all the possible actions that can be sampled to generate the new graph. It also embraces invalid actions, so the model must learn to set zero probability for this. The APD is calculated for all graphs present in the training set in the preprocessing phase. There are three probable actions, the probability of adding a new node (*f*_add_), the probability of connecting the last node in the graph to another existing one (*f*_conn_), and the probability of finishing the graph (*f*_fin_). All these probabilities must sum to one for each graph and are the target vectors to be learned by the model in the training phase. The APD is the output of the model.

The combination of the GGNN, the message passing phase, with the global readout block is translated through the equations presented below, the calculus structure behind the system. The GGNN is defined by eqs 1–5 and is represented in the system by the functional form, eqs 6–10.^11,22^
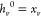
1
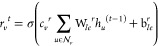
2
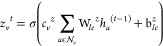
3
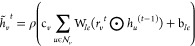
4
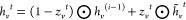
5where *h*_*v*_^0^ is the node feature vector for the initial node *v* at the GGNN layer and is equal to its node feature vector in the graph; *r*_*v*_^*t*^ is a GRU’s gate in the specific MLP layer, *t*, and relative to the node *v*; σ is the sigmoid function; c_*v*_ = *c*_*v*_^*z*^ = *c*_*v*_^*r*^ =  are normalization constants;  is the set of neighbor nodes for *v*; *u* is a specific node in the graph;  is a trainable weight tensor in *r* regarding the edge label, ; *b* is a learnable parameter; *z* is also a GRU’s gate; ρ is a non-linear function; and ⊙ is an element-wise multiplication.
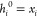
6
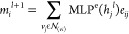
7
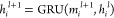
8
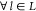
9where *m*_*i*_^*l*+1^ and *h*_*i*_^*l*+1^ are the incoming messages and hidden states of node *vi*, respectively; e_*ij*_ is the edge feature vector for the edge connecting *v*_*i*_ and *v*_*j*_; *l* is a GNN layer index; and *L* is the final GNN layer index.
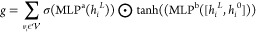
10where *g* is the final graph embedding.

The global readout block is translated by eqs 11–16, presented below.^11^ The activation function of the block is the SoftMax function, which converts a vector of numbers into a vector of probabilities. As a generalization of the sigmoid function, this function is largely applied in SciML to normalize weighted sum value outputs, so the probabilities sum to one.^23^
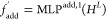
11
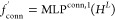
12
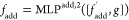
13
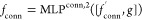
14
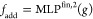
15
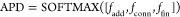
16

The training phase of this system, GGNN and global readout block, is executed in mini batches. The activation function of the model is the scaled exponential linear unit (SELU), presented in eqs 17 and 18, which is applied after every linear layer in the MLP.^24^ The model training loss is given by the Kullback–Leibler divergence between the target APD and predicted APD. Kullback and Leibler (1951)^25^ introduced the Kullback–Leibler divergence as a measure of discrepancy between probabilities based on information.^26^
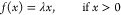
17
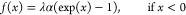
18where α = 1.6733 and λ = 1.0507.

Moreover, all the models use the Adam optimizer. Introduced by Kingma and Ba (2017),^27^ the Adam optimizer is a straightforward first-order gradient-based optimization algorithm. This optimization function carries out the sparse gradients and non-stationary objectives. The Adam-defined parameters are presented in Supporting Information, Table S1.^28^

The training models are evaluated by sampling graphs in established intervals of epochs. During this step, the evaluation metrics are calculated using the generated graphs of this phase, Table 2. The uniformity-completeness Jensen-Shannon divergence (UC-JSD) is one of the evaluation metrics presented. This metric is related to the Kullback–Leibler divergence and its application to an average distribution.^29^ In this work, the UC-JSD calculates the distribution of negative log-likelihood per sampled action in each set.

**Table 2:** Evaluation Metrics

metrics	description
PV	percentage of valid molecules in the set
PU	percentage of unique molecules in the set
PPT	percentage of molecules that were finished through sampling of finish action
PVPT	percentage of valid molecules in the set of PPT molecules
ν_av_	average number of nodes per graph in the set
ε_av_	average number of edges per node
UC-JSD	uniformity-completeness Jensen–Shannon divergence

A molecule is considered valid if the total count of hydrogens to be added is according to the type of atoms, explicit bonds, and formal charges of the molecule. After the addition of the hydrogens, if they are incompatible, the molecule can still be edited to solve this problem. The edition is through the RDKit function rdkit.Chem.SanitizeMol().^30^ The function verifies valences, set aromaticity, hybridization, and molecule conjugation. If one of the analyses fails, the molecule is modified to solve the problem. If the sanitizing fails, an error is raised, and the molecule is considered invalid. If the invalid molecule is one of the output constituents, it is represented as “[Xe]”.

The final phase is the generation. The graphs and the output APD are given as the input. During this step, the sampled actions imply the growth of the new graph and choices, such as the kind of atom to add. Moreover, the graph construction can be finished if the sampled action is to finish or if an invalid action occurs. The invalid actions are the addition of a new node in a node that does not exist in the graph. Connecting nodes that are already connected and adding a node in a graph that already has the maximum number of nodes are also invalid actions. Furthermore, the hydrogens are ignored during the training and generation phases. They are added according to the atoms’ valency in the generated graphs.

Each graph goes through the system one by one; the growth is carried out node by node or edge by edge until it is finished and given as an output. The model’s training stops, and the number of defined molecules to be generated is given as an output according to the convergence criteria of the training loss, defined as three significant figures.

The structure of the model’s architecture is defined through hyperparameters. These variables are set as a means to guide and direct the training and performance of the SciML model.^31^ Also, they can be divided into two categories: algorithm and model parameters. The algorithm parameters consist of tuning parameters encircling the number of epochs, the learning rate decays, momentum, and the learning rate. At the same time, the model parameters are composed of variables such as the number of layers, layer type, number of neurons, and activation function.^32^

The definition of these hyperparameters has major implications for the methodology’s accuracy. In this work, they were defined through a sensitivity analysis and can be found in the Supporting Information of this article, Table S2.

## 3 Results

**Figure 4:**
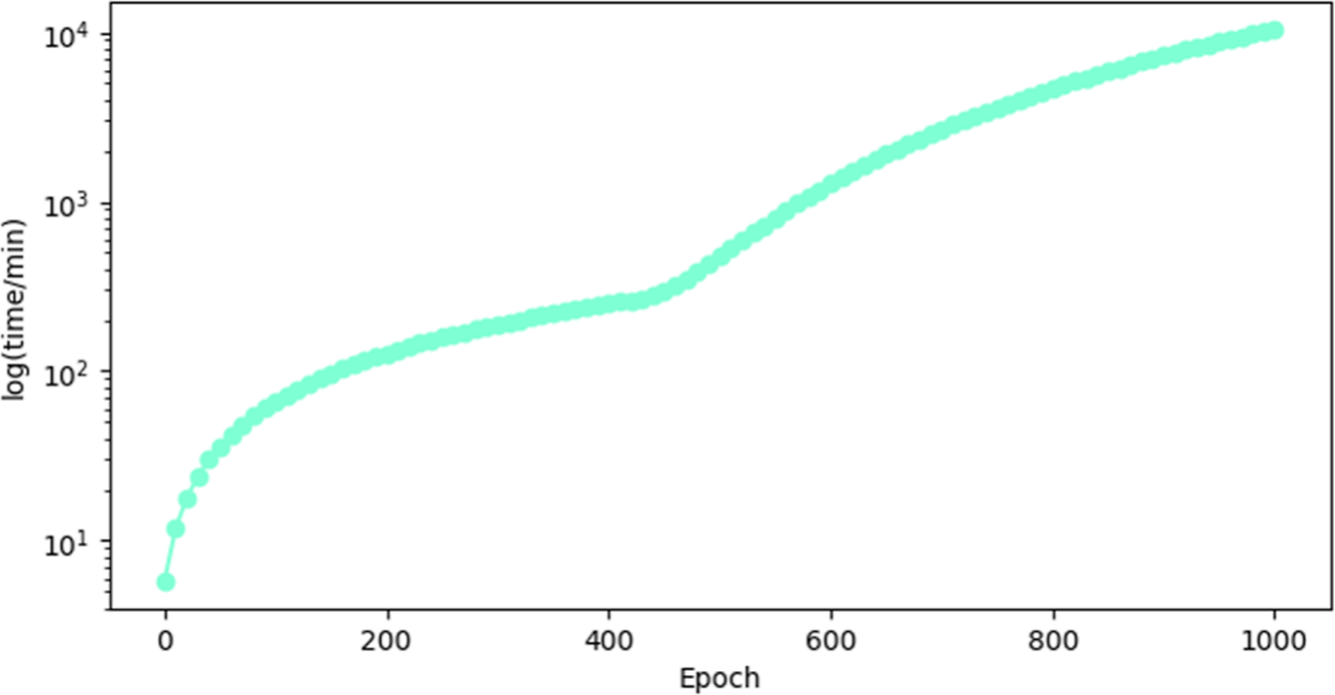
Logarithm of time in minutes for each training epoch.

The DGM was trained for 1000 epochs. The training was performed in a Linux environment, in a server, through a VirtualBox installed in a Windows 10 system. The server has an AMD Ryzen 9 5900X 12-Core Processor 3.79 GHz; 32.0 GB of installed RAM; an operative system of 64 bits; and an NVIDIA GeForce RTX 3060 GPU. The Oracle VM VirtualBox has an Ubuntu 64-bit operative system. Within these conditions, the time required to train the neural network is presented in Figure 4, in which the logarithm of the time in minutes is represented for each training epoch.

In Figure 4 it is possible to verify that the required time to train the neural network is approximately 11,000 min, which corresponds to 7 days and 15 h of training. Considering these computational costs for training a model, the hyperparameters’ optimization was carried out through sensitivity analysis. In this way, finding a good model within a reasonable computational effort was possible.

The epoch that presented the best results was chosen based on the minimization of the UC-JSD values and the average likelihood of training, validation, and generation. Epoch 780 was defined as the generation epoch, as it presented the minimal UC-JSD between all the epochs (UC-JSD of −0.0216).

**Figure 5:**
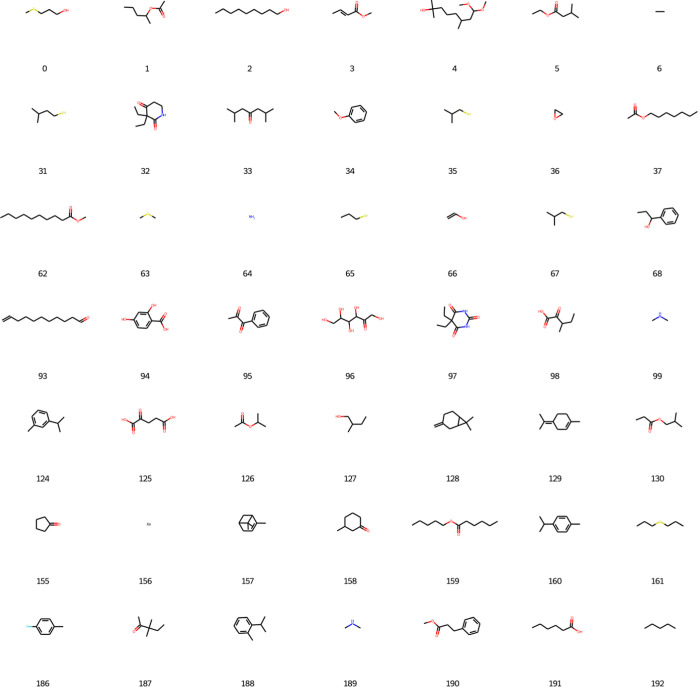
New designed molecules from DGM part 1.

**Figure 6:**
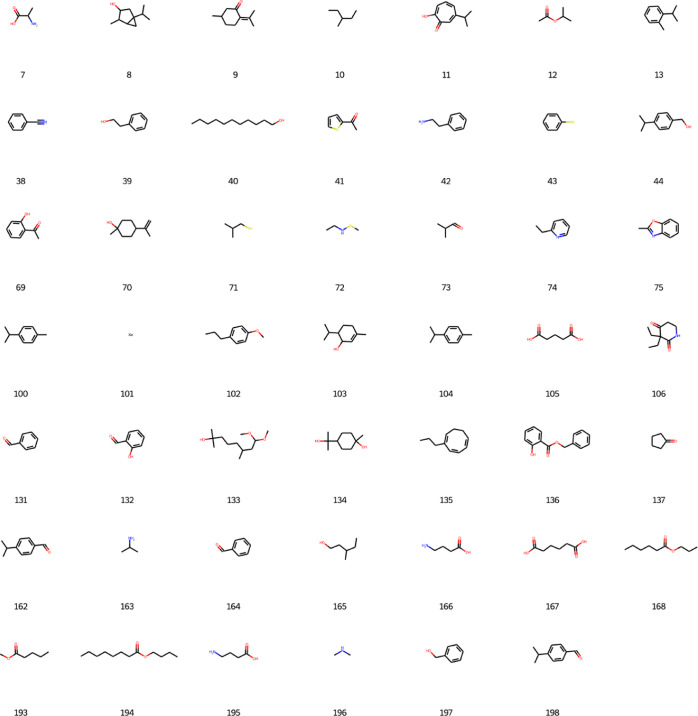
New designed molecules from DGM part 2.

Furthermore, 200 newly designed molecules were generated based on the epoch 780, and 197 of those were considered valid by the network. The molecules are presented in Figures 5 and 6.

**Figure 7:**
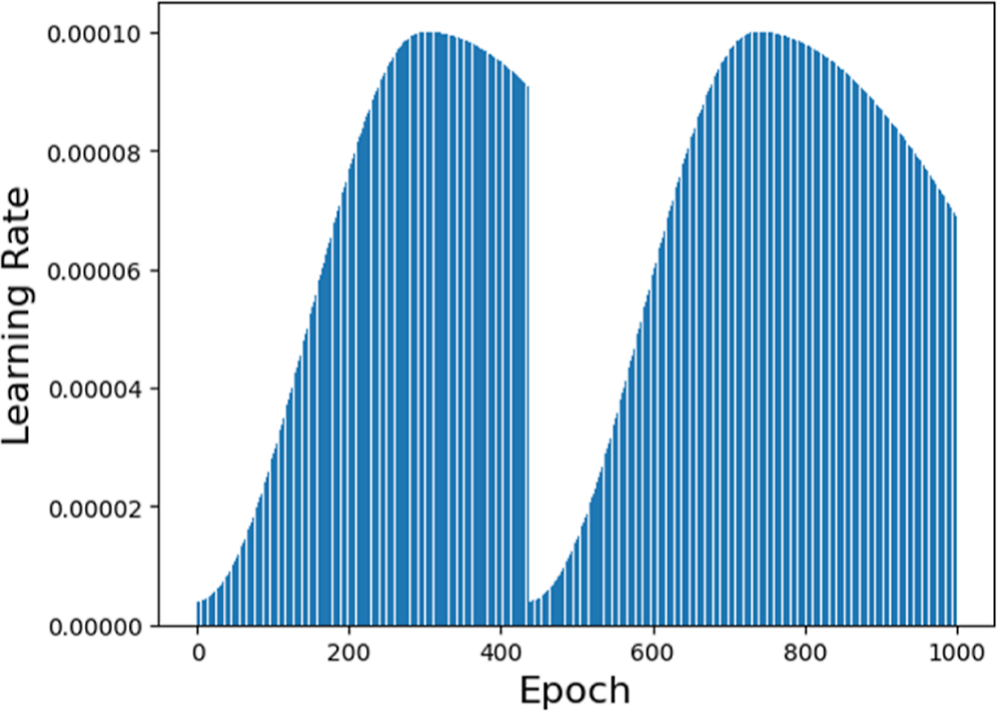
Generative model’s learning rate.

The data treatment and graphs presented in this work were implemented in Google Colab notebook in Python. The results for the learning rate are presented in Figure 7. It is possible to visualize that the generation epoch presents a high value for the learning rate, but it is not the highest value. This notice is important because the higher the value of the learning rate, the more biased is the neural network’s prediction. However, the lower is the value, the more overfitted is the neural network.

**Figure 8:**
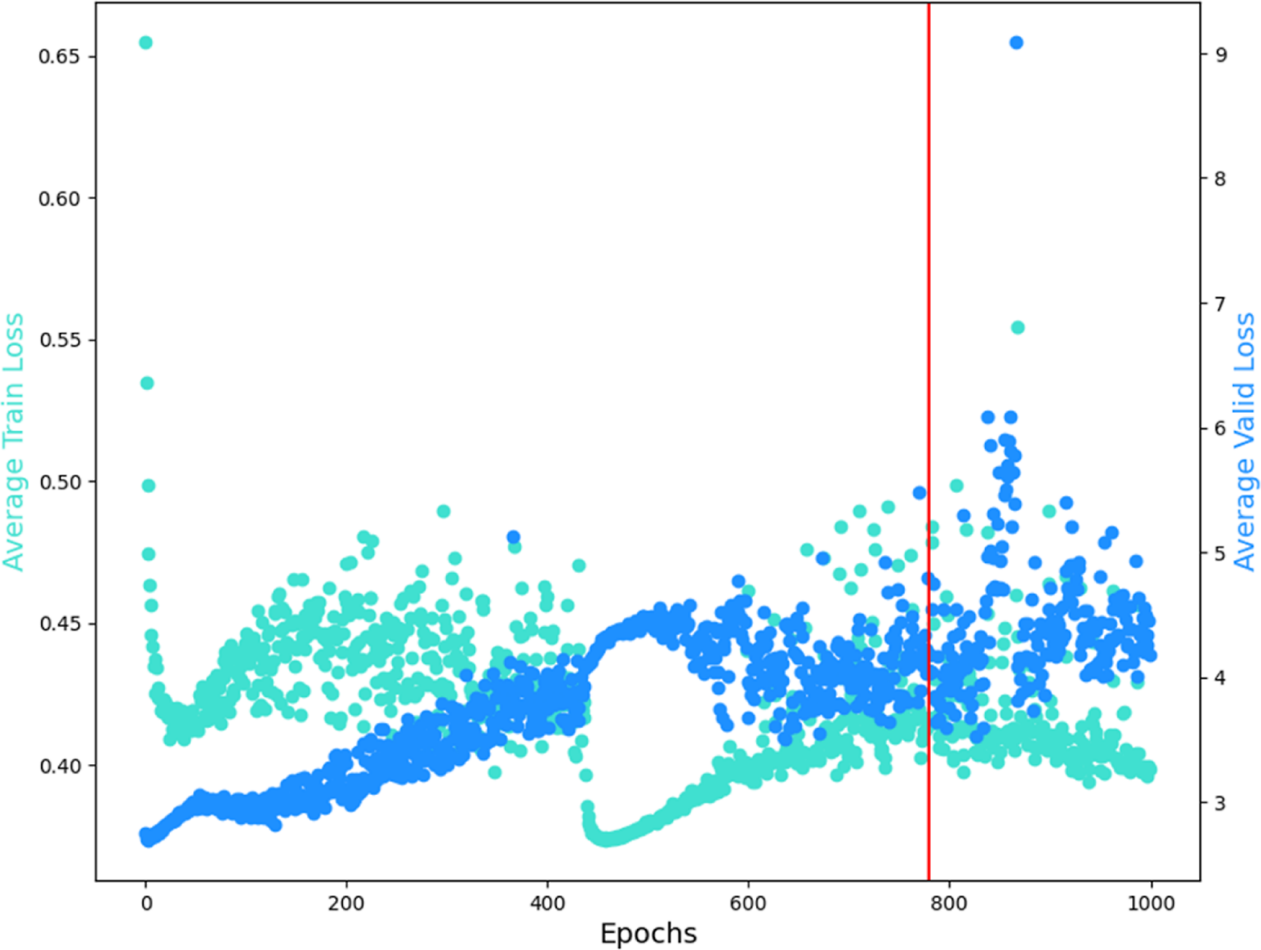
Average train and valid loss for the 1000 training epochs.

In order to analyze the convergence with respect to the number of epochs, a graph to compare the average train loss and average valid loss for each epoch is presented in Figure 8, in which the data represented by the color blue denote the average valid loss, the data represented by the color cyan denote the average train loss, and the red line is the result for the generative epoch chosen. The training loss is analyzed to evaluate the data fitting of the model. It is calculated by the sum of the errors in each graph in the training set. Meanwhile, the validation loss is analyzed to evaluate the model’s performance on the validation set. It is calculated in the same way as the training loss, i.e., it sums the errors for each graph in the validation set.

In Figure 8 it is possible to verify that from epoch 0 to 500, the average train loss decreases quickly, then increases, and starts slowing decreasing. In contrast, the average valid loss increases slowly, implying a risk of overfitting. However, from epoch 500 to 800, the average train loss and valid loss present a good fitting. From epoch 900 to 1000, it is possible to visualize an increase of the average valid loss relative to the average train loss, while the average train loss starts decreasing. There is another symptom of overfitting. In this case, the best solution is to stop the training a previous epoch where a better performance was observed. Based on this analysis, the generation epoch should be chosen between the epochs 500 and 800.

Based on the convergence and on the percentage of valid molecules in the set (PV), percentage of unique molecules in the set (PU), percentage of molecules that were finished through the sampling of finish action (PPT), and percentage of valid molecules in the set of PPT molecule (PVPT) metrics, the generation epoch chosen was the 780. The convergence and evaluation results for the generation epoch are presented in Table 3.

**Table 3:** Convergence and Evaluation Results

epoch	780
average likelihood per molecule in validation	26.04
average likelihood per molecule in training	1.85
average likelihood per molecule in generation	0.32
UC-JSD	–0.02
learning rate	9.90 × 10^–^^5^
average train loss	0.41
average valid loss	4.05
PV (0-1)	1.00
PVPT (0-1)	1.00
PPT (0-1)	1.00
run time/s	129228.44
ν_av_	8.35
ε_av_	1.96
PU (0-1)	0.95

**Figure 9:**
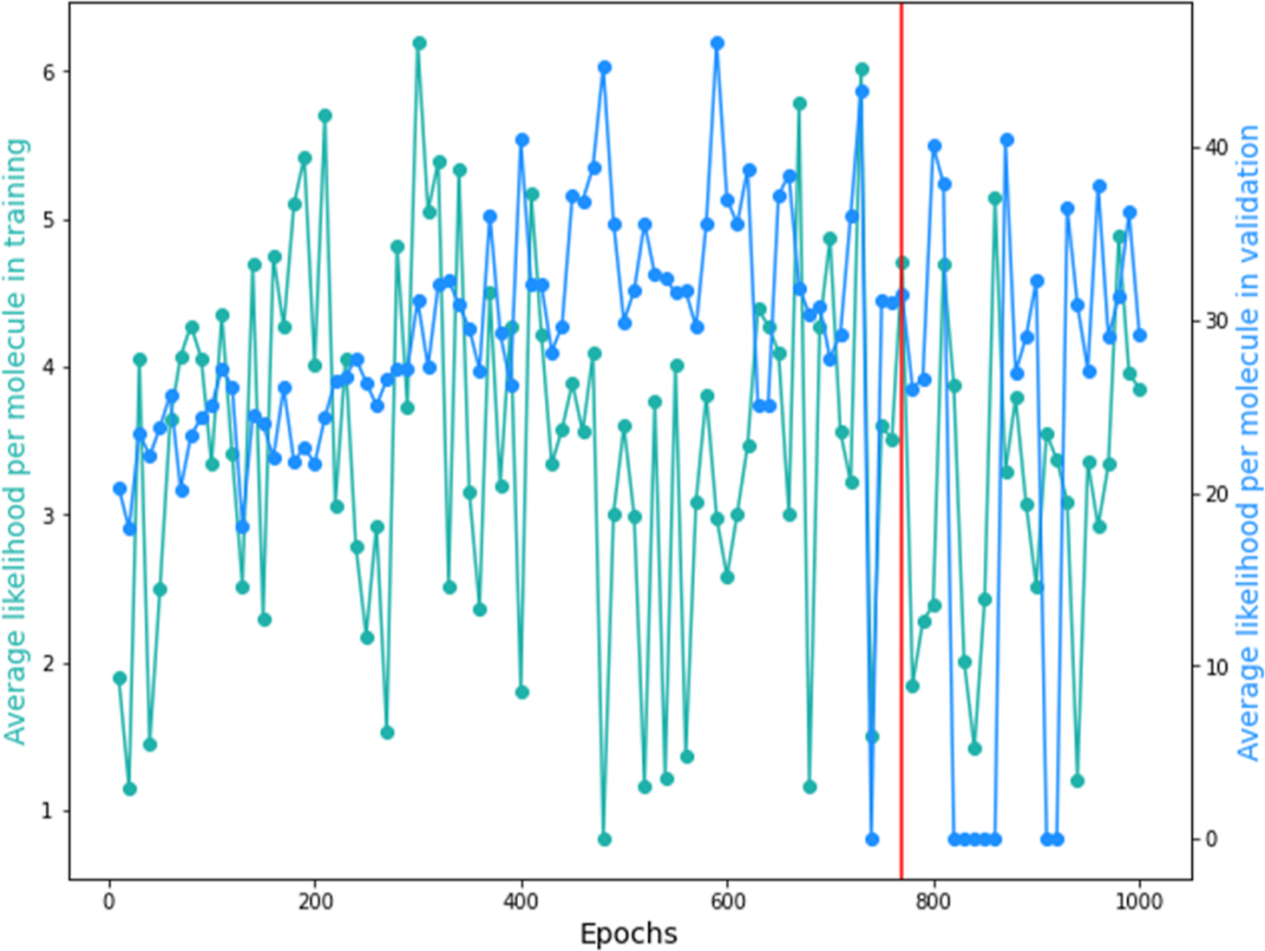
Average likelihood per molecule in training and in validation for the 1000 training epochs.

In order to have a better visualization of the obtained results from the DGM during training and validation, Figure 9 presents the average likelihood per molecule in training and validation. In this case, the data represented by the color blue denote the average likelihood per molecule in validation, the data represented by the color green denote the average likelihood per molecule in training, and the red line represents the result for the generative epoch chosen. The average likelihood metric is analyzed to verify the train and validation performance, to obtain information on how likely it is to obtain a data set as the original gave as input. Having that in mind, the higher the value of likelihood is, the better the fit of the model is. In Figure 9, it is possible to visualize that the chosen generative epoch does not present the highest average likelihood for the training and the validation. However, it is one of the highest points of the average likelihood for both training and validation. It was considered good enough regarding all the selected metrics for the generative epoch, as mentioned when discussing Figure 8 and Table 3.

**Figure 10:**
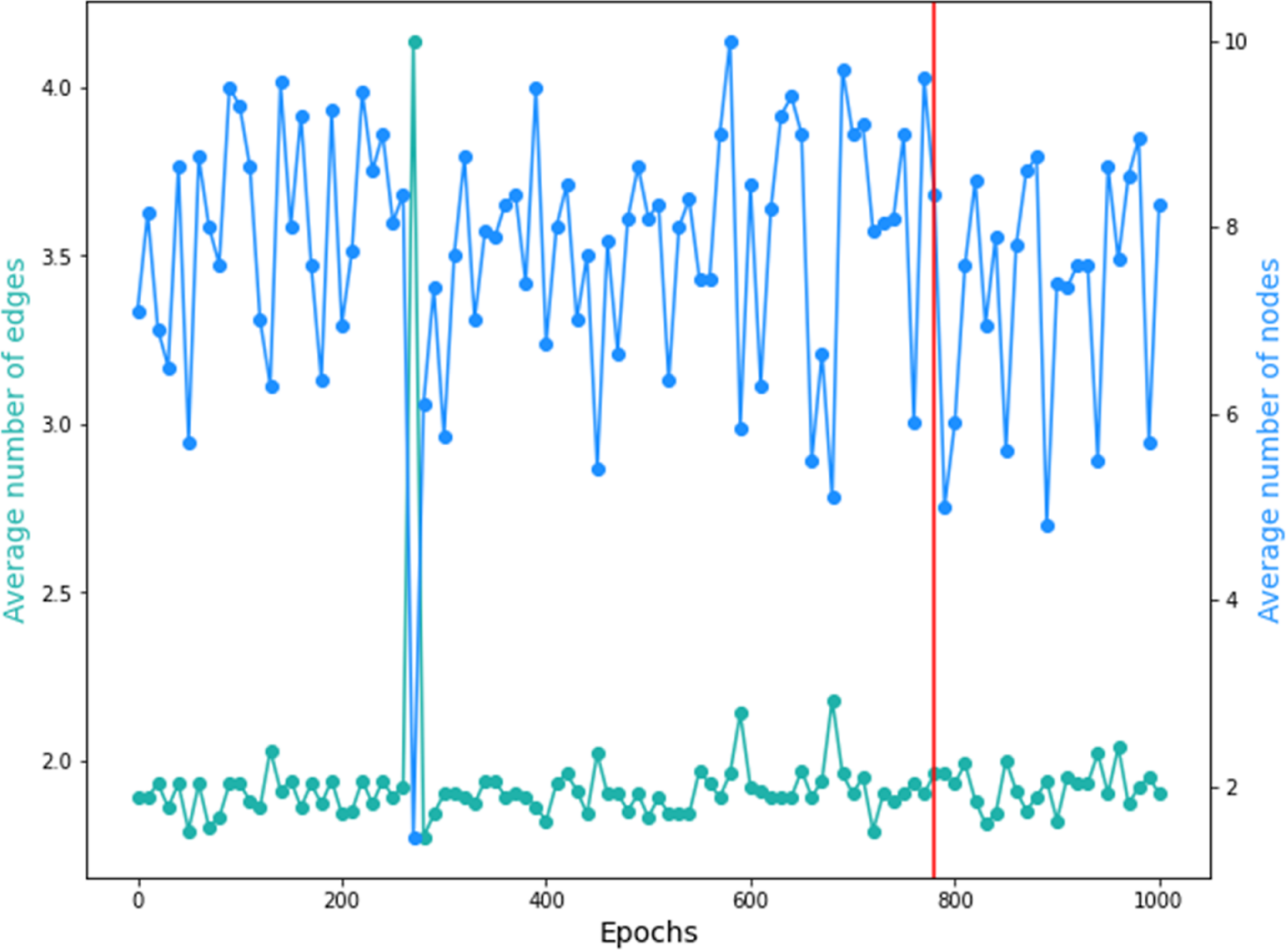
Average number of edges and nodes for the 1000 training epochs.

The analyses of the generation results regarding the chemical structure of the obtained molecules can be carried out through the visualization of Figure 10. In this figure, the data represented by the color blue denote the average number of nodes in the resulting graphs, representing atoms in the molecular structure; the data represented by the green color denote the average number of edges in the resulting graphs, representing the bonds per atom in the molecular structure; and the vertical red line highlights the resulting average number of nodes and edges per node in the resulting graphs of the generative epoch chosen. It is possible to visualize that the average number of nodes ranges between three and four nodes per graph, while the average number of edges per node is around two. For the generative epoch, the average number of nodes and edges per node is between the common range for all the epochs in the training, not presenting an outlier. However, it is important to notice that the average is calculated counting invalid molecules. So, this analysis is only performed to verify the presence of outliers and if the epochs’ results are congruent within themselves.

To evaluate the obtained results, the 200 molecules generated were studied and analyzed. The general results regarding the number of molecules that are valid, invalid, existent, non-existent, already used in the flavor industry, and not used in the flavor industry are presented in Table 4.

**Table 4:** Generated Molecule Assessment Results

categories	number of molecules	percentage of molecules (%)
valid molecules	197	98.5
invalid molecules	3	1.5
existent	195	97.5
non-existent	2	1
used in the flavor industry	155	77.5
not yet used in the flavor industry	40	20
toxic	5	2.5

As already mentioned, the generation based on the training epoch 780 obtained 197 valid molecules. Considering those 197 molecules, 2 of them, even though they are considered valid and have canonical SMILES, are not recognized by the ChemSpider^33^ and PubChem^34^ online databases. The non-existent molecules SMILES are shown in eqs 19 and 20.
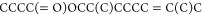
19
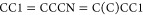
20

It is important to notice that the generative model proposed throughout this work has been implemented in order to obtain and design molecules to be applied in the flavor industry. However, it does not imply that the generated molecules do not already exist or that they are not already employed in other industrial sectors. Considering the 200 molecules obtained through SciML, only 1% do not have a defined reaction path or exist in the online databases. This result shows that the generative model can be confidently applied to obtain molecules to compose flavor-based products, not necessarily requiring to be newly synthesized.

When studying the molecules obtained through the generation model regarding the application in the flavor industry, 77.5% of them are already employed as flavoring agents or flavor enhancers. The remaining 20% of valid molecules that are not yet employed in the focused sector must be studied, and their chemical structure must be analyzed to be considered for the “new role” of the flavoring agent. Actually, 15% of them can be considered as a new approach to a flavoring agent, while the other 5% are toxic molecules. Concerning the toxic molecules, the five molecules obtained are classified as carcinogenic. Meanwhile, the percentage of molecules that are not yet employed in the flavor industry is composed of molecules used in the pharmaceutical industry, package production, lubricants, solvents, other flavors’ precursor, or perfumes. As an example, three of the obtained molecules that are in this 15% are going to be analyzed in the following paragraphs.

**Figure 11:**
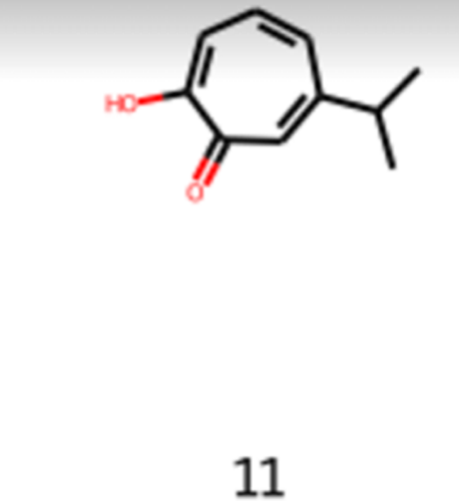
Image obtained as the output of the generative model of the 2-hydroxy-6-propan-2-ylcyclohepta-2,4,6-trien-1-one.

The first molecule to be analyzed is the 2-hydroxy-6-propan-2-ylcyclohepta-2,4,6-trien-1-one (CAS number: 499-44-5), also known as Hinokitiol, shown in eq 21 and Figure 11. It is a natural molecule that is found in a traditional Japanese tree, Taiwanese ninoki tree, used in the pharmaceutical industry to regulate iron transport in animals. It is also applicable to prevent infections, as an antistatic, as a fragrance component, and in hair conditioning products. This molecule is already used as a food additive in Japan. However, in the literature, it is not possible to find information about it being used in the flavor industry worldwide. It is an interesting molecule to be analyzed and considered to be applied in the flavor industry considering the pharmacological properties, the vast application in different industries sectors, the fact that it is possible to be naturally extracted from *Cupressaceae* family’s trees, and that it was already approved as not carcinogenic in Canada.^35,36^
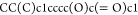
21

**Figure 12:**
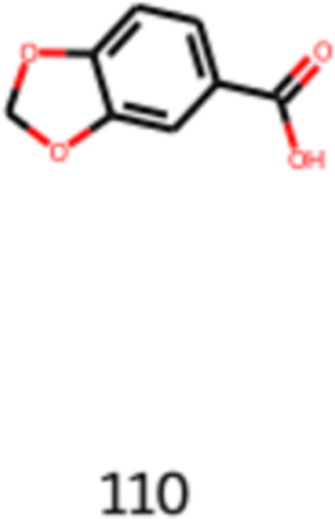
Image obtained as the output of the generative model of 1,3-benzodioxole-5-carboxylic acid.

The following obtained molecule to be analyzed is the 1,3-benzodioxole-5-carboxylic acid (CAS number: 94-53-1), also known as methyprylon, shown in eq 22 and Figure 12. This molecule is used in the cosmetic industry for skin conditioning and protection. It can be naturally extracted from the *Nectandra amazonum* and *Pongamia pinnata* var. *pinnata*, plants from tropical biome which are used in medicine. This molecule has antifungal and skin healing properties. It is possible to visualize in Figure 12 that this molecule has functional groups that are common in flavored molecules, such as ether and carboxylic acid groups. Considering the industrial applications and properties, 1,3-benzodioxole-5-carboxylic acid is an interesting molecule to be further studied and has its toxicology assessed in order to consider it to be applied in the flavor industry.^37^
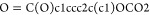
22

**Figure 13:**
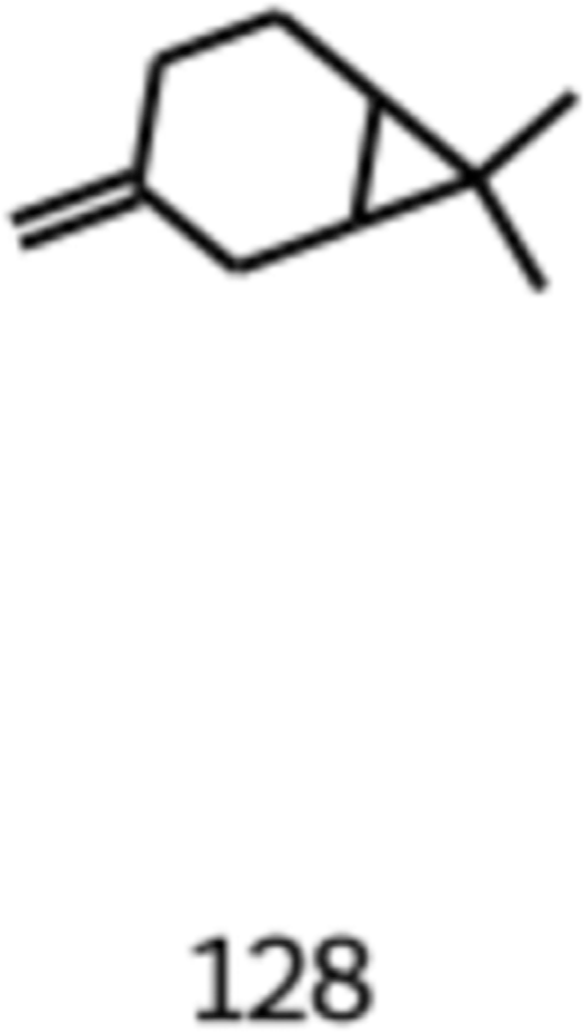
Image obtained as the output of the generative model of 7,7-dimethyl-3-methylene-bicyclo[4.1.0]heptane.

Finally, the third molecule to be analyzed is the 7,7-dimethyl-3-methylene-bicyclo[4.1.0]heptane (CAS number: 554-60-9), also known as β-carene, shown in eq 23 and Figure 13. This molecule can be extracted from the essential oil of Algerian cypress. It is considered a volatile compound found in herbs and has been the focus of studies regarding the use of natural products for food preservation and in the analysis of under-utilized herbs. The carene molecule is used in the perfume industry. However, the β-carene could not be found in the literature as a perfume component or flavoring agent. Considering the studies performed and the natural aspect of the molecule, it is important to consider a further analysis of properties and toxicology to evaluate the application of this molecule in the flavor industry.^38^
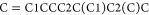
23

**Figure 14:**
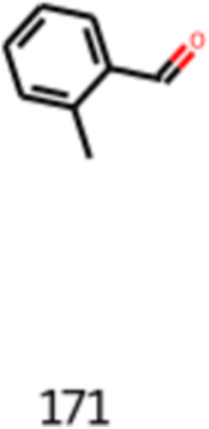
Image obtained as the output of the generative model of 2-methylbenzaldehyde.

Another relevant aspect of the obtained molecules that are valid and not yet applied in the flavor industry is the obtained isomeric structures of molecules already applied in the flavor industry. An example is the 2-methylbenzaldehyde (CAS number: 529-20-4), also known as *o*-tolualdehyde, shown in eq 24 and Figure 14. This molecule is used as a fragrance compound. However, as a flavoring agent, it is the *p*-tolualdehyde that is used. The *p*-tolualdehyde has a floral, sweet, and spicy flavor. Considering the flavor industry application of the molecule’s para isomer, it is interesting to analyze the possibility of using the ortho isomer as well, having in mind that the reaction in the human body can be different for different isomers.
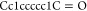
24

It is possible to conclude that even though the generative model designs the molecules through random sampling of actions, it can find molecules that are already used in the flavor industry or in other industrial. Meanwhile, it was possible to verify that the new approach to develop flavors and flavor-based products does not necessarily imply discovering and trying new synthesis paths to obtain new molecules. Actually, it is possible to discover molecules already available in the market, some of them largely applied in other industry sectors, that can be studied and analyzed to fulfill flavor engineering needs. Alternatively, it is also possible to obtain through the generative model suggestions of molecules that are already applied in the flavor engineering field as flavoring agents or flavor enhancers and can be considered in flavor-based product development.

## 4 Conclusions

This work launches a new standpoint in flavor engineering based on SciML. The main goal was to generate new flavored molecules that could be synthesized and applied in the industry to develop flavor-based products, hence addressing an increasing challenge found in the flavor industry.

The methodology consisted of a generative framework development to generate new flavor molecules based on a database extracted from FlavorDB’s website. The proposed method was able to design several molecules to be applied in the flavor industry. The results demonstrate the overall concept proposed in this work and its potential to help in flavor design.

This work is focused on a methodology development to generate flavored molecules. These generated molecules can be evaluated concerning their existence or not in the market and whether they can be easily synthesized or not. If they already exist in the market but are not used in flavor-based products yet, the search for a synthesis route is not required, and the bureaucracy of compound regulation could be easier. To address this issue and demonstrate this concept, a few of the generated molecules were analyzed and their availability in the market is shown.

## Supporting Information Available

Table of PyTorch default parameters’ values for the Adam optimizer and table of DGM hyperparameters (PDF)
