# Supplementary material for: Generating Flavor Molecules Using Scientific Machine Learning
Source: ACS Omega. 2023 Mar 15;8(12):10875–87. doi: 10.1021/acsomega.2c07176 (PMC10061502; doi:10.1021/acsomega.2c07176)
Supplement: Supplementary file 1 — ao2c07176_si_001.pdf [file ao2c07176_si_001.pdf]

Generating flavor molecules using Scientific Machine Learning

Luana P. Queiroz [1,2], Carine M. Rebello[3], Erbet A. Costa[3], Vinícius V. Santana[1,2], Bruno C. L. Rodrigues[1,2], Alírio E. Rodrigues[1,2], Ana M. Ribeiro[1,2] and Idelfonso B. R. Nogueira[4,*]

1 LSRE-LCM - Laboratory of Separation and Reaction Engineering – Laboratory of Catalysis and Materials, Faculty of Engineering, University of Porto, Rua Dr. Roberto Frias, 4200-465 Porto, Portugal;

2 ALiCE - Associate Laboratory in Chemical Engineering, Faculty of Engineering, University of Porto, Rua Dr. Roberto Frias, 4200-465 Porto, Portugal;

3 Departamento de Engenharia Química, Escola Politécnica (Polytechnic Institute), Universidade Federal da Bahia, Salvador 40210-630, Brazil;

4 Chemical Engineering Department, Norwegian University of Science and Technology, Sem Sælandsvei 4, Kjemiblokk 5, Trondheim, Norway;

To whom correspondence should be addressed: idelfonso.b.d.r.nogueira@ntnu.no*

Supporting Information

Table S1 - Pytorch default parameters' values for Adam optimizer.

Parameters	Learning rate	Betas	Eps	Weight decay	AMSGrad	Maximize
**Default values**	1.00 x 10^{-3}	(0.90, 0.99)	1.00 x 10^{-8}	0.00	False	False

The defined hyperparameters are presented in the following table, Table S2.

Table S2 - Deep generative model hyperparameters.

Parameters	Deep Generative Model's Value
α	-
Batch size	20
Block size	1000
Epochs	1000
Generation epoch	790
GGNN activation function	SELU
GGNN depth	4
GGNN dropout probability	0
GGNN hidden dimension	250
GGNN width	100
Initial learning rate	1.00x10^{-4}
Learning rate decay factor	0.99
Learning rate decay interval	10
Loss function	Kullback-Leibler divergence
Maximum relative learning rate	1.00
Message passing layers	3

Message size – input size of GRU	100
Minimum relative learning rate	$1.00\text{x}10^{-4}$
MLP activation function	SoftMax
MLP depth (Layers 1 and 2)	4
MLP dropout probability (Layers 1 and 2)	0
MLP hidden dimension (Layers 1 and 2)	500
Number of samples	200
Optimizer	Adam
σ	-
Weight decay	0
Weight initialization	Uniform
